# Implication of IZUMO2 in the cell‐in‐cell phenomenon: A potential therapeutic target for triple‐negative breast cancer

**DOI:** 10.1111/1759-7714.15189

**Published:** 2024-01-23

**Authors:** Toshiya Higashi, Chiemi Saigo, Wakana Chikaishi, Hirokatsu Hayashi, Yuki Hanamatsu, Manabu Futamura, Nobuhisa Matsuhashi, Tamotsu Takeuchi

**Affiliations:** ^1^ Department of Gastroenterological Surgery and Pediatric Surgery Gifu University Graduate School of Medicine Gifu Japan; ^2^ Department of Pathology and Translational Research Gifu University Graduate School of Medicine Gifu Japan; ^3^ The United Graduate School of Drug Discovery and Medical Information Sciences Gifu University Gifu Japan; ^4^ Center for One Medicine Innovative Translational Research; COMIT Gifu University Gifu Japan; ^5^ Department of Breast Surgery Gifu University Hospital Gifu Japan

**Keywords:** adiponectin‐expressing Tregs, cell‐in‐cell activity, IZUMO2, monoclonal antibody, triple‐negative breast cancer

## Abstract

**Background:**

Triple‐negative breast cancer (TNBC) is characterized by the loss of estrogen receptor, progesterone receptor, and human epidermal growth factor receptor 2. The aggressive clinicopathological features and resistance to currently available therapeutics of the disease warrant an urgent need for the development of novel alternate therapeutic options. We have previously reported adiponectin‐expressing regulatory T cells (A‐Tregs), which can induce apoptosis in TNBC through the cell‐in‐cell phenomenon. In this study, we aimed to elucidate the molecule that allows TNBC cells to engulf A‐Tregs.

**Methods:**

A monoclonal antibody, which repressed the engulfment of A‐Tregs by TNBC cells, was developed. Immunoprecipitation followed by mass spectrometry and small interfering RNAs‐mediated gene silencing was performed to characterize the antigen.

**Results:**

We successfully generated a monoclonal antibody, designated G1D7, which abrogated the engulfment of A‐Tregs by TNBC and subsequent A‐Treg‐mediated apoptosis. G1D7 detected the immunoglobulin‐like type I membrane protein IZUMO2, a molecule related to IZUMO1 that is essential for cell–cell membrane binding and fusion of sperm to oocyte.

**Conclusion:**

The findings highlight the importance of IZUMO2 on TNBC cells in facilitating the cell‐in‐cell phenomenon by A‐Tregs.

## Graphical abstract

(a) Several adiponectin‐expressing Tregs attached to MDA‐MB‐231 cells (red signal). Note the integration of adiponectin‐expressing Tregs into the cytoplasm (yellow, merging of red and green). (b and c) An angle of 45 degrees and vertical cell‐in‐cell images are shown in (b) and (c), respectively. Note the destruction (denoted by the white line) of MDA‐MB‐231 cells after the occurrence of the cell‐in‐cell phenomenon. (d) An antibody to IZUMO2, designated G1D7 (final concentration of 1 μg/mL) markedly reduced the occurrence of cell‐in‐cell phenomenon. (e) siRNA‐mediated silencing of the *IZUMO2* gene also abrogated the cell‐in‐cell phenomenon.

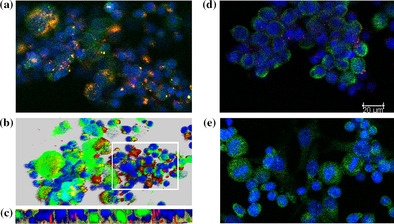

## INTRODUCTION

Patients with triple‐negative breast cancer (TNBC) experience unfavorable prognosis owing to the aggressive clinicopathological features and the lack of validated molecular targets for the disease.^1^ Therefore, the development of novel alternate therapeutic options for patients with TNBC is urgently required.^2^

Adiponectin, a well‐characterized insulin‐sensitizing adipokine, induces autophagic cell death^3^ and apoptosis via fatty acid metabolic reprogramming in breast cancer.^4^ Insufficient amount of adiponectin followed by obesity is believed to lead to mammary carcinogenesis.^5^ Furthermore, a major receptor of the of active form of adiponectin, T‐cadherin (also known as H‐cadherin or CDH13)^6^, ^7^, ^8^, ^9^ is frequently lost in breast cancer because of hypermethylation of its promoter region.^10^, ^11^, ^12^ Accordingly, it is critical to develop an efficient adiponectin‐vehicle to enable the clinical application of adiponectin for patients with breast cancer.

In addition to adipose tissues, several tissues such as the colon, ovaries, salivary glands, liver, and skeletal muscle express adiponectin.^13^ Additionally, a population of T regulatory cells (Tregs) residing within the complex of thymic nurse cells also expresses adiponectin.^14^, ^15^, ^16^ These adiponectin‐expressing Tregs have promising therapeutic implications in TNBC based on their ability to induce apoptosis through the cell‐in‐cell phenomenon.^17^, ^18^

In our previous studies, we have successfully developed a murine adiponectin‐expressing Treg, designated A‐Treg, from an experimental thymic tumor model.^17^, ^18^ Notably, A‐Tregs exhibited the cell‐in‐cell phenomenon and subsequently induced apoptosis of TNBC cells in vitro^17^ and decreased tumor volume in an orthotopic murine TNBC model.^18^ These findings indicate that A‐Tregs could be an effective adiponectin vehicle for regulating TNBC. However, the molecular mechanism by which A‐Tregs recognize and invade TNBC cells to exhibit the cell‐in‐cell phenomenon is unclear. As a complementary analysis, assessing the expression status of the molecule expressed in TNBC and involved in facilitating the cell‐in‐cell activity of A‐Tregs could serve as a predictive tool to determine the potential effect of A‐Tregs against TNBC. In the present study, we aimed to elucidate the molecule expressed in cancer cells that facilitates the infiltration of A‐Tregs into these cancer cells.

## METHODS

### Cells and culture

The MDA‐MB‐157^19^ and 231^20^ TNBC cell lines were obtained from the American Type Culture Collection (Manassas). The mesothelioma cell line MPM‐1 was established and maintained in our laboratory.^21^ A‐Tregs were established and characterized as previously described.^15^, ^16^, ^17^, ^18^ Cells were cultured in Dulbecco's modified Eagle medium‐high glucose (4500 mg/L; Sigma‐Aldrich) with 10% fetal bovine serum. Cells were passaged for no more than 6 months after resuscitation.

### Generation of monoclonal antibodies

The experimental protocol was approved by the Animal Care Committee of Gifu University Graduate School of Medicine, Gifu, Japan (approval nos. 2020–066, 2021–149, and 2022‐087). Briefly, a BALB/c mouse was intraperitoneally immunized every week with 1 × 10^7^ MPM‐1 cells. Monoclonal antibodies were generated according to the modified method of Köhler and Milstein.^22^, ^23^ Hybridoma clones were screened using a two‐step process, as indicated below, and cloned via limiting dilution. Initially, we selectively identified clones producing antibodies that exhibit reactivity with the surface membrane of MPM‐1 cells through the immunofluorescent staining procedure detailed below. Subsequently, we examined whether the candidate antibodies could suppress the cell‐in‐cell activity of A‐Tregs infiltrating TNBC cells in vitro. Subclasses of antibodies were determined using an Isotyping kit (Antagen Pharmaceuticals) following the manufacturer's instructions. The antibody was purified from culture supernatants using Immuno‐Assist MG‐PP (Kanto Chemical).

### Immunofluorescence staining

Immunofluorescence staining was performed as previously described.^24^ Briefly, cells were incubated with antibodies for 30 min at 4°C. After washing, the cells were incubated with Alexa Fluor 488 goat anti‐mouse IgG (H + L) (cat. no. A11001; Invitrogen) for 30 min at 4°C. The cells were analyzed using a Guava EasyCyte cell analyzer (Cytek Biosciences).

The occurrence of the cell‐in‐cell phenomenon was evaluated using a confocal laser scanning microscope (Leica TCS SP8; Leica Biosystems) as previously described.^17^ Briefly, A‐Tregs were labeled using the LuminiCell Tracker670‐Cell labeling kit (Sigma‐Aldrich) according to the manufacturer's instructions. We employed cytokeratin immunostaining using anti‐pan‐cytokeratin antibody, clone AE1/AE3 (Leica Biosystems), followed by incubation with Alexa Fluor 488 goat anti‐mouse IgG (H + L) for visualizing the cytoplasm of MDA‐MB‐231 breast cancer cells.

### Immunoprecipitation, peptide mass fingerprinting, and mass spectrometry

The procedure was performed as previously described.^23^ Briefly, the purified G1D7 antibody was bound to M‐270 epoxy magnetic beads (Dynabead Antibody Coupling Kit; Life Technologies) according to the manufacturer's protocol. The G1D7‐bound protein band from MDM‐MB‐231 cell lysates was digested with trypsin and subjected to matrix‐assisted laser desorption ionization time‐of‐flight analysis (Microflex LRF 20; Bruker Daltonics). Spectra were acquired at 300 shots per spectrum over an m/z range of 700–4000 and calibrated through two‐point internal calibration using trypsin auto‐digestion peaks (m/z 842.5099, 2211.1046). The peak list was generated using the Flex Analysis 3.0 software. The threshold used for peak‐picking was as follows: a minimum resolution of monoisotopic mass was set at 500, and the signal‐to‐noise ratio (S/N) was established at 6. The search program MASCOT, developed by Matrixscience (http://www.matrixscience.com/), was used for protein identification via peptide mass fingerprinting. The following parameters were used for the database search: trypsin as the cleaving enzyme, a maximum of one missed cleavage, iodoacetamide (Cys) as complete modification, oxidation (Met) as partial modification, monoisotopic masses, and a mass tolerance of ±0.2 Da.

### Small interfering RNA (siRNA)‐mediated RNA interference

siRNA‐mediated silencing of target genes was performed as previously described.^23^ We employed siRNAs to silence the *IZUMO2* gene (cat. no. AM16708, Assay IDs 139 737 and 139 738; Thermo Fisher Scientific, Inc.), while a Trilencer‐27 Universal scrambled negative control siRNA‐duplex (OriGene) was used as a nonsilencing control. The siRNAs were transfected into cells using lipofectamine RNAiMAX (Invitrogen) following the manufacturer's instructions. The cells were used for subsequent studies 72 h after transfection.

### Quantitative real‐time reverse transcription polymerase chain reaction (RT‐qPCR)

cDNA synthesis from the total RNA and subsequent PCR were performed using an RT‐PCR kit (Takara) as previously described.^25^ Real‐time PCR was performed on a LightCycler (Roche Diagnostics GmbH) using the SYBR green reaction kit (Roche Diagnostics) according to the manufacturer's instructions. The following primers were used for real‐time RT‐PCR: IZUMO2‐forward 5′‐ CCGCCATGCCTCTGGCTTTGACCCTTCTGC‐3′; IZUMO2‐reverse 5′‐ CTC CAT GCCCATCAGCACGGCCCCGGCGCG‐3′; GAPDH‐forward 5′‐ GAAGGTGAAGGTCGGAGTC‐3′; GAPDH‐reverse 5′‐GAAGATGGTGATGGGATTTC‐3′.

The expression of each target gene was analyzed using the 2^−ΔΔCT^ method described by Livak and Schmittgen.^26^ The ΔCT values were normalized to that of GAPDH in both the Trilencer‐27 Universal scrambled negative control siRNA‐treated (control) and si‐IZUMO2‐treated groups. The values for the si‐IZUNO2‐treated group were then calculated for each target gene as the fold change relative to the control group (control; set to 1.0).

### Immunoblotting

Immunoblotting was performed as described by Towbin et al. with modifications as previously described.^27^, ^28^, ^29^ Cell lysates were electrophoresed on sodium dodecyl sulfate‐polyacrylamide gels and electroblotted onto polyvinylidene difluoride membranes (Immobilon‐FL Transfer Membrane; Millipore). The membrane was blocked with Block Ace (blocking milk; Yukijirushi) and subsequently incubated with 0.5 μg/mL G1D7 and rabbit anti‐glyceraldehyde‐3‐phosphate dehydrogenase (GAPDH) antibody (cat no. G9545; Sigma‐Aldrich). For fluorescent immunodetection, we employed goat anti‐rabbit IgG highly cross‐adsorbed secondary antibody Alexa Fluor Plus 800 (cat. no. A32735; Invitrogen; Thermo Fisher Scientific Inc.) and goat anti‐Mouse IgM (Heavy chain) secondary antibody, Alexa Fluor 647 (cat. no. A‐21238, Invitrogen; Thermo Fisher Scientific Inc). Fluorescent signals were detected using an Invitrogen iBright 1500 gel imaging system (Thermo Fisher Scientific Inc).

## RESULTS

### Generation of a monoclonal antibody, designated G1D7, which inhibited the cell‐in‐cell activity of A‐Tregs to TNBC cells

**FIGURE 1:**
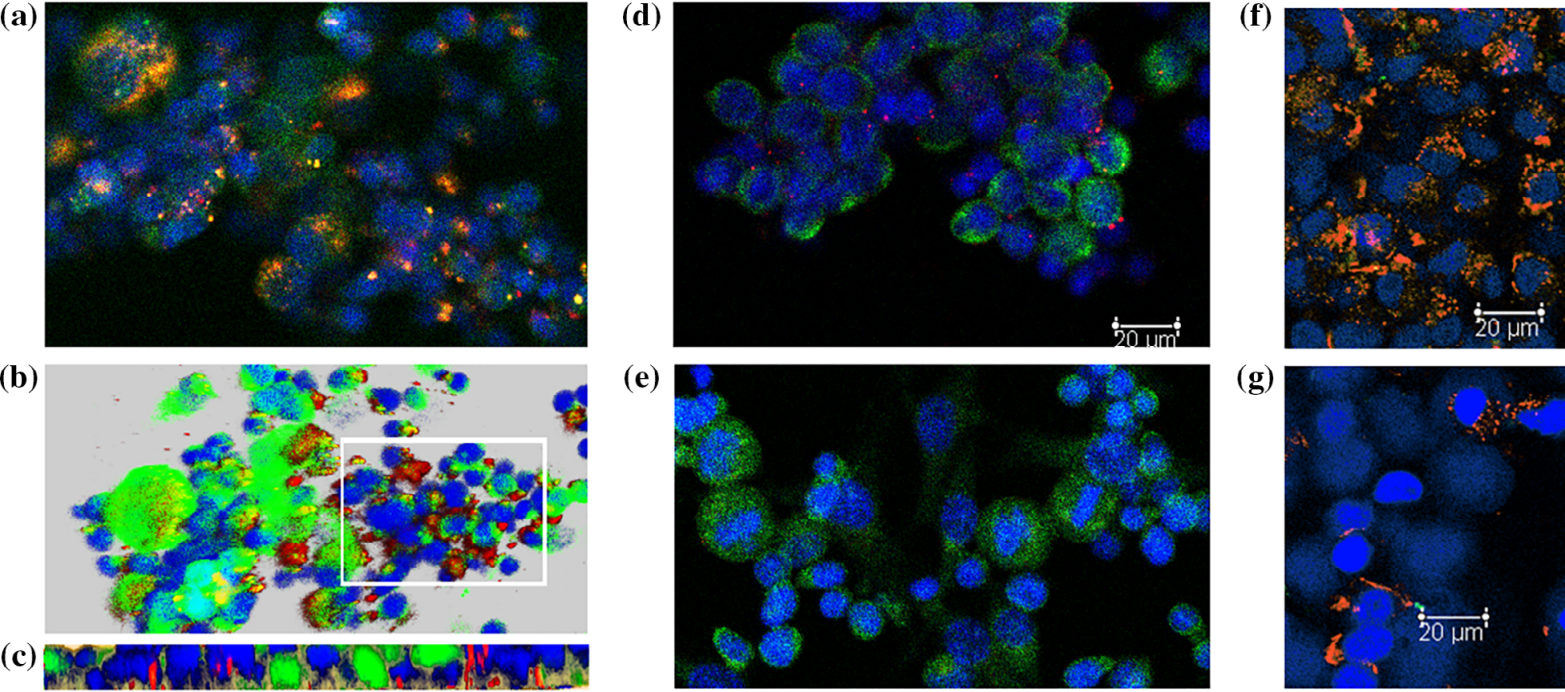
The G1D7 antibody or siRNA‐mediated silencing of the *IZUMO2* gene impaired the cell‐in‐cell phenomenon of A‐Tregs within TNBC cells. A‐Tregs were labeled using a LuminiCell Tracker670‐cell labeling kit and detected as red by confocal laser imaging, while the cytoplasm of MDA‐MB‐231 TNBC cells was stained with pan‐cytokeratin antibody and detected as green. After 16 h of coculture of A‐Tregs and MDA‐MB‐231 cells, the cells were washed, fixed, and nuclear‐stained with DAPI. A‐Tregs heavily attached to MDA‐MB‐231 cells are shown in red, and the cytoplasmic integration in MDA‐MB‐231 cells (i.e., the cell‐in‐cell phenomenon of A‐Tregs) is shown in yellow (merging of red and green). (a) Several adiponectin‐expressing Tregs attached to MDA‐MB‐231 cells (red signal). Note the integration of adiponectin‐expressing Tregs into the cytoplasm (yellow, merging of red and green). (b and c) An angle of 45 degrees and vertical cell‐in‐cell images are shown in (b) and (c), respectively. Note the destruction (denoted by the white line) of MDA‐MB‐231 cells after the occurrence of the cell‐in‐cell phenomenon. (d) G1D7 (final concentration of 1 μg/mL) markedly reduced the occurrence of cell‐in‐cell phenomenon. (e) siRNA‐mediated silencing of the *IZUMO2* gene also abrogated the cell‐in‐cell phenomenon. Similar results were obtained using another TNBC cell line, MDA‐MB‐157 (f, Mock; g, G1D7) without cytokeratin staining. Scale bar: 20 μm.

A preliminary experiment showed that A‐Tregs exhibited marked cell‐in‐cell activity to a mesothelioma MPM‐1 cell. Accordingly, we employed MPM‐1 as an immunogen to generate monoclonal antibodies, which inhibited the cell‐in‐cell activity of A‐Tregs in cancer cells. One antibody, which was composed of μ and κ chains, appeared to inhibit the cell‐in‐cell activity of A‐Tregs in MDA‐MB‐157 and MDA‐MB‐231 TNBC cells and was designated G1D7. A representative result is shown in Figure 1.

### G1D7 recognized the immunoglobulin superfamily protein IZUMO2

Subsequently, we purified the G1D7 antigen from MDA‐MB‐231 cells using G1D7‐binding M‐270 epoxy magnetic beads. An approximately 20‐kDa protein band, which was also detected via immunoblotting using G1D7, was observed using G1D7‐binding M‐270 epoxy magnetic beads but not the control murine IgM‐binding M‐270 epoxy magnetic beads. This 20‐kDa band was analyzed via peptide mass fingerprinting, and it appeared to be the human IZUMO2 protein, with a notably high Mowse score.

**FIGURE 2:**
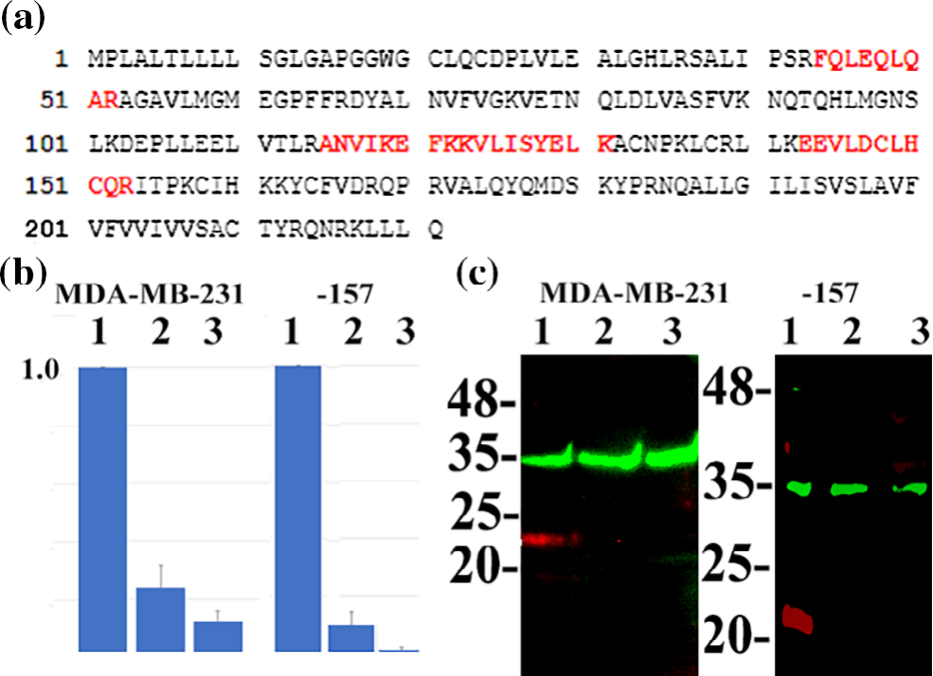
Identification of IZUMO2 as a recognition protein by G1D7. (a) Peptide coverage is present in human IZUMO2. Matched peptides are shown in red. (b) Quantitative RT‐PCR showed that siRNA‐mediated silencing using #139737 (lane 2) or #139738 (lane 3) siRNA‐duplex successfully reduced the mRNA expression of *IZUMO2* in MDA‐MB‐231 and MDA‐MB‐157 cells compared with mock siRNA‐duplex treatment (Trilencer‐27 Universal scrambled negative control siRNA‐duplex; lane 1). The mean and standard deviation is shown (*n* = 3) (c) Fluorescent immunoblotting results showing that siRNA‐mediated silencing of *IZUMO2* resulted in the reduction of the red G1D7 protein band (1, mock siRNA; 2, #139737 siRNA; 3, #139738 siRNA) in MDA‐MB‐231 and MDA‐MB‐157 cells. The green band represents the GAPDH protein.

Subsequently, we performed siRNA‐mediated silencing of the *IZUMO2* gene in MDA‐MB‐231 cells, which resulted in the reduction of the 20‐kDa G1D7 band, as observed in the immunoblotting analysis. We conclude that G1D7 demonstrates specificity toward the IZUMO2 protein. Representative data are shown in Figure 2.

siRNA‐mediated downregulation of *IZUMO2* abrogated the cell‐in‐cell activity of A‐Tregs within MDA‐MB‐231 cells (Figure 1).

## DISCUSSION

We have reported the cell‐in‐cell phenomenon of A‐Tregs derived from thymic T cells that were originally maintained in coculture with thymic stromal cells. These Tregs spontaneously expand with an ability to promote cell death in TNBC cells both in vitro and in vivo.^15^, ^16^, ^17^, ^18^ In this study, we observed that in TNBC, the expression of IZUMO2, a protein structurally related to IZUMO1, was important for the cell‐in‐cell activity of A‐Tregs, subsequently leading to A‐Treg‐mediated TNBC apoptosis.

IZUMO1 is localized on the acrosomal membrane and translocates to the sperm plasma membrane during acrosome reaction.^30^ Mammalian sperm‐egg adhesion is believed to depend on the transinteraction between the sperm‐specific IZUMO1 and oocyte‐specific GPI‐anchored receptor JUNO.^31^ A reduction in fusion occurs when sperm are treated with antibodies against IZUMO1,^30^ and artificially expressed IZUMO1 induces cell‐to‐cell fusion in cultured somatic cells.^32^ These findings indicate that IZUMO1 acts as a fusogen that is physiologically restrictively expressed in sperm cells during fertilization.

IZUMO2 is a type I transmembrane protein that contains an N‐terminal domain known as the IZUMO domain.^33^ Although the pathobiological properties of IZUMO2 are largely unclear, anti‐IZUMO2 antibodies were reported be found in 11 of 25 serum samples from immunoinfertile women but in none of the serum samples from fertile women.^34^ Further extensive studies are needed to ascertain whether IZUMO2 can also act as a fusogen through its IZUMO domain in TNBC cells to exhibit the cell‐in‐cell phenomenon with A‐Tregs.

Previously, a cell‐in‐cell phenomenon was reported to be exhibited by the Treg cell line HOZOT toward several cancer cells. HOZOT cells are derived from human umbilical cord blood, cocultured with mouse stromal cells, and are characterized as a cytotoxic Treg line exhibiting cell‐in‐cell activity.^35^, ^36^, ^37^, ^38^ The presence of Tregs with cell‐in‐cell activity has also been reported in hepatocellular carcinoma.^39^ Based on the present findings, we hypothesize that the A‐Tregs as well as other related Treg subtypes known to exhibit cell‐in‐cell activity might recognize IZUMO2 or its related molecules on partner cells for cell fusion.

In conclusion, our study revealed a novel function of IZUMO2 on TNBC cells as a putative fusogen in the cell‐in‐cell phenomenon with A‐Tregs. Studies to determine whether the expression status of IZUMO2 may also could be used as a potent therapeutic indicator for the adoptive transfer of A‐Tregs in patients with TNBC are also ongoing.

## AUTHOR CONTRIBUTIONS

All authors read and approved the final manuscript. Conceptualization, C.S. and T.T.; Methodology, T.H.; Investigation, T.H., W.C., H.H., Y.H., and C.S.; Formal Analysis, M.F., N.M., and C.S.; Resources, C.S. and T.T.; Writing—Original Draft, C.S.; Writing—Review & Editing, T.T.; Visualization, C.S.; Supervision, T.T.; Funding Acquisition, C.S. and T.T.; Data Curation, C.S.

## CONFLICT OF INTEREST STATEMENT

The authors report no potential conflict of interest.
